# Efficacy of Psycho-Cardiology therapy in patients with acute myocardial infarction complicated with mild anxiety and depression

**DOI:** 10.3389/fcvm.2022.1031255

**Published:** 2023-01-26

**Authors:** Xiaoliang Chen, Mengya Zeng, Chen Chen, Dan Zhu, Li Chen, Zuying Jiang

**Affiliations:** ^1^The Affiliated Chenzhou Hospital, Hengyang Medical School, University of South China, Chenzhou, China; ^2^The Second Affiliated Hospital of Hainan Medical University, Haikou, Hainan, China

**Keywords:** Psycho-Cardiology, pharmacological intervention, acute myocardial infarction, anxiety, depression

## Abstract

**Objective:**

To evaluate the efficacy of Psycho-Cardiology therapy among patients with acute myocardial infarction (AMI) complicated with mild anxiety and depression.

**Methods:**

Two hundred and fifty-six patients with AMI who were admitted to the Cardiovascular Department of Chenzhou First People's Hospital from January 2018 to January 2020 were selected as subjects, and randomly divided into the control group (*n* = 128) and the Psycho-Cardiology treatment group (*n* = 128). Prior to the intervention, the general clinical data of the enrolled patients, such as gender, age, comorbidities (hypertension, diabetes) and smoking history, were compared, which revealed no statistical differences between the two groups (*P* > 0.05). The control group was given routine treatments such as reperfusion and secondary prevention of coronary heart disease, while the treatment group was given Psycho-Cardiology intervention in addition to the aforementioned treatments.

**Results:**

No significant differences in PHQ-9 and GAD-7 scores were observed between the control and treatment groups at admission (*P* > 0.05). After the Psycho-Cardiology treatment, the PHQ-9 and GAD-7 scores of the treatment group decreased significantly. Based on the 1-year post-treatment comparison, the left ventricular ejection fraction was improved more significantly in the Psycho-Cardiology treatment group, showing statistical significance (*P* < 0.05). The treatment group exhibited statistically significantly low incidences of adverse cardiovascular events, such as recurrent angina pectoris, heart failure, malignant arrhythmia, recurrent myocardial infarction and death (*P* < 0.05).

**Conclusions:**

Psycho-Cardiology therapy is remarkably efficacious in improving the anxiety, depression, cardiac function and reducing the occurrence of adverse cardiovascular events, which can better improve the long-term prognosis of patients with AMI compared to the traditional treatments.

## 1. Introduction

With the development of social economy and the improvement of people's living standards, coronary atherosclerotic heart disease (CHD) has become a common, multiple and serious disease. Acute myocardial infarction (AMI), a common form of coronary heart disease, occurs at a younger age and is acute and severe, threatening human health. The survival rate of patients with acute myocardial infarction has been improved by medication and/or surgery (1–3). However, patients with acute myocardial infarction have problems such as decreased working ability and need to take secondary preventive drugs for coronary heart disease for life. These factors lead to different types of psychological problems in some patients. Clinically, anxiety and depression disorders are more common. More and more studies have shown that patients with coronary heart disease are prone to anxiety and depression, which can reduce the quality of life of patients (4–6). Anxiety, depression and other negative emotions not only seriously lower the quality of life of patients, but also lay a hidden danger for the recurrence of coronary heart disease and other cardiovascular adverse events (7). Because patients are often not good at expressing symptoms other than coronary heart disease, similar psychological and emotional disorders are often not easy to be paid attention to by clinical workers. Nekouei et al. (8) found that compared with simple medical treatment, comprehensive treatment combined with psychological intervention not only actively treats the underlying diseases, but also has a significant effect on the treatment of post-PCI patients' negative emotions such as depression.

“Psycho-Cardiology,” also known as behavioral cardiology, is a discipline studying the emotional, sociobehavioral and environmental issues related to heart disease (9). This discipline is mainly based on cardiovascular disease, which is characterized by the inclusion of patients' mental and psychological disorders into the whole heart disease protection system. In 1998, 38 authoritative experts in the field of psychology and cardiology held a conference, the first to reach a relevant consensus, since then more experts continue to improve the intervention measures of bicardiac medicine, further promoting the leapfrog progress of this discipline (10). Many studies (9, 11, 12) have pointed out that psychological intervention on the basis of conventional drug therapy can not only promote the recovery of patients with underlying diseases, but also improve the prognosis of patients and improve the quality of life. Hence, this study evaluates the application value of “Psycho-Cardiology model” among AMI patients complicated with mild anxiety, in order to provide a theoretical basis for clinical treatment.

## 2. Materials and methods

### 2.1. Clinical data

A total of 256 subjects were studied, all of whom were AMI patients admitted to our hospital from January 2018 to January 2020. They were grouped with the random group generator. For 128 patients in the control group, routine treatments such as reperfusion therapy (thrombolytic/interventional) and secondary prevention of coronary heart disease were given. Meanwhile, 128 patients in the Psycho-Cardiology treatment group were given psychotherapy along with routine treatments and, if necessary, anxiolytics and antidepressants were also given. This study was approved by our hospital's Medical Ethics Committee. No significant differences were found between the two groups regarding clinical data like gender, age, comorbidities (diabetes, hypertension) or smoking history (*P* > 0.05), which were clinically comparable.

Inclusion criteria: (1) age ≤ 85 years (age range of the entire subjects: 38–85 years); (2) compliance with the 2017 ESC Guidelines on the Diagnosis of AMI (13), confirmed by ECG, dynamic ECG, echocardiography, coronary angiography and other examinations; (3) capable of communicating normally and giving informed consent; (4) NYHA cardiac functional class: I–III; (5) PHQ-9 and GAD-7 scores ranging between 5–9.

Exclusion criteria: (1) refused to participate in this study. (2) Cognitive dysfunction, bipolar disorder and other serious mental disorders; inability to cooperate; past history of depression, organic brain syndrome or use of certain psychoactive substances. (3) Those with severe hepatic and renal insufficiency. (4) Pregnant women; patients with severe cardiac insufficiency, congenital heart disease, valvular disease, aortic dissection; patients with tumors, severe cerebrovascular diseases, infectious diseases.

### 2.2. Methods

Control group: conventional treatments like reperfusion therapy (thrombolytic/interventional) and secondary prevention of coronary heart disease were given, as well as health education.

Medical treatment group: adjuvant psychotherapy was given on the basis of aforementioned conventional treatments. (1) Psychotherapy: we often communicated with patients by means of listening, companionship and encouragement. Meanwhile, psychoanalytic therapy (i.e., free association and other methods) was used to analyze unconscious behaviors of these patients. By exploring the patients' life history, we tried to understand how they experienced the development and change of life, in order to help them better cope with the present and future life. (2) Behavioral therapy: by introducing the clinical features, pathogenesis, treatment hints and prognosis of coronary heart disease, patients were helped to establish a healthy and correct knowledge-based view of disease, and to understand the significance of smoking cessation, as well as the effects of rational diet and moderate exercise on the prognosis, in order to change unhealthy behaviors and correct bad habits. The patients were advised to give more companionship to and communication with family members in their life, and try to avoid being alone. (3) Exercise therapy: cardiac rehabilitation training was given under the doctors' guidance, gradually achieving a certain exercise tolerance. (4) Relaxation therapy: the ward environment was kept quiet, and some music was played depending on the patients' psychological mood. When any patient had plentiful mood swings and easily got nervous and excited, some soothing light music was played to purify their mind. When any patient was depressed or anxious, some upbeat music was played, or a comedy video on the ward TV. The patients were guided to relax, focus on the music or video, and imagine the beauty of nature based on music. After playing the music for a while, the patients' muscles could relax completely. There were no patients with moderate or severe anxiety and depression, so drug intervention was not used in the study. The follow-up duration was 1 year.

### 2.3. Evaluation indicators

In this study, the psychological state of patients in the two groups before and after treatment was evaluated on PHQ-9 and GAD-7 (14, 15). The 9-item PHQ-9 score was rated on a 5-point scale as follows: normal (< 5 points), mild (5–9 points), moderate (10–14 points), moderate-to-severe (15–19 points), and severe (20–27 points). The 7-item GAD-7 score was rated on a 4-point scale as follows: normal (< 5 points), mild (5–9 points), moderate (10–14 points), and severe (15–21 points).

### 2.4. Statistical analysis

The data involved in this study were all analyzed in Excel using the SPSS 25.00 (Chicago, IL, USA) software. Among the experimental data, the enumeration data was χ^2^, while the measurement data was *T*. The statistical significance of intra-group indicators was defined by 0.05. Statistical analysis was performed using SPSS 25.00. The continuous variables between two groups were compared by the independent samples *t*-test, and the results were expressed as means ± SDs. The categorical variables were expressed as percentages and analyzed by the chi-square test. *P* values of < 0.05 were considered statistically significant.

## 3. Results

### 3.1. Inter-group comparison of clinical data

The general clinical data of the patients in the control and treatment groups at admission, such as gender, age, comorbidities (hypertension, diabetes), and smoking history, were compared, which found no statistical differences between the two groups in Table 1 (*P* > 0.05).

**Table 1:** Inter-group comparison of clinical data [*n* ($\bar{x}\;\pm \;s$)].

**Item**	**Control group** **(*n* = 128)**	**Psycho-cardiology treatment group** **(*n* = 128)**	**χ^2^**	**P-value**
Age (years)	60.48 ± 9.21	61.05 ± 9.73	0.481	0.631
**Gender**				
Male/female	102/26	101/27	0.024	0.877
NSTEMI/STEMI	31/97	32/76	0.471	0.492
Smoking history	82 (64.06%)	85 (66.41%)	0.155	0.694
Diabetes	41 (32.03%)	36 (28.13%)	0.464	0.496
Hypertension	58 (45.31%)	69 (53.91%)	1.891	0.169

### 3.2. Inter-group comparison of PHQ-9 and GAD-7 anxiety/depression scores before and after treatment

Prior to treatment, no differences in PHQ-9 or GAD-7 scores were noted between the two groups (*P* > 0.05). After treatment, the observation group exhibited better scores than the control group (*P* < 0.05), as shown in Table 2.

**Table 2:** Inter-group comparison of PHQ-9 and GAD-7 scores before and after treatment [*n* ($\bar{x}\;\pm \;s$)].

**Group/Item**	**PHQ-9 score**		***t***	**P-value**	**GAD-7 score**		***t***	**P-value**
	**Before treatment**	**After treatment**			**Before treatment**	**After treatment**		
Observation group (*n* = 128)	6.61 ± 1.33	5.41 ± 1.26	14.113	0.000	7.13 ± 1.26	6.08 ± 1.46	11.553	0.000
Psycho-Cardiology treatment group (*n* = 128)	6.65 ± 1.31	4.51 ± 1.80	22.694	0.000	7.09 ± 1.23	5.14 ± 1.22	23.409	0.000
*t*	0.141	5.616			0.283	5.976		
*P*	0.821	0.010			0.654	0.000		

### 3.3. Inter-group comparison of LVEF before and after treatment

After 1-year follow-up, echocardiography re-examination was performed to compare the left ventricular ejection fraction (LVEF) between the two groups before and after treatment. More significant improvement was noted in the Psycho-Cardiology treatment group, showing statistical significance (*P* < 0.05), as shown in Table 3.

**Table 3:** Inter-group comparison of LVEF before and after treatment [*n* ($\bar{x}\;\pm \;s$)].

**Group/item**	**LVEF%**		***t***	**P-value**
	**Before treatment**	**After treatment**		
Observation group (*n* = 128)	45.91 ± 4.04	49.16 ± 4.71	15.595	0.000
Psycho-cardiology treatment group (*n* = 128)	45.66 ± 4.06	50.32 ± 4.66	21.086	0.000
*t*	0.570	1.989		
*P*	0.569	0.048		

### 3.4. Adverse cardiovascular events during 1-year follow-up

Compared to the observation group, the Psycho-Cardiology treatment group exhibited lower incidences of adverse cardiovascular events like recurrent angina pectoris, heart failure, malignant arrhythmia, recurrent myocardial infarction and death (*P* < 0.05), as shown in Table 4.

**Table 4:** Inter-group comparison of adverse cardiovascular events before and after treatment (*n*, %).

**Group/item**	**Recurrent angina pectoris**	**Heart failure**	**Malignant arrhythmia**	**Recurrent myocardial infarction**	**Death**
Observation group (*n* = 128)	26	35	31	16	25
Psycho-Cardiology treatment group (*n* = 128)	15	20	18	6	11
*P*	0.042	0.022	0.039	0.026	0.023

## 4. Discussion

AMI is a common life-threatening heart disease, which is prone to symptoms such as angina pectoris, heart failure, and malignant arrhythmia in the later stage. Anxiety and depression are highly prevalent psychiatric disorders, which affect more than 300 million patients worldwide. In addition, they have long been associated with an increased risk of cardiovascular disease (16). The prevalence of depression in patients with cardiovascular disease is as high as 15%−30% (17), which is two to three times higher than that in the general population. Numerous studies have also shown that patients with depression have a 60% higher risk of cardiovascular disease compared with healthy controls (18). The early manifestations of AMI are persistent chest tightness and chest pain, even combined with a sense of near-death. Intense physical discomfort can lead to negative emotions such as tension, anxiety, and depression. A large number of studies (19–22) have shown that physical discomfort caused by major diseases usually coexists with emotional disorders and even mental illnesses. Diseases are often interconnected, and cardiovascular disease and psychological problems are no exception. However, clinical work often ignores the psychological problems of patients with myocardial infarction, resulting in an increased incidence of adverse cardiovascular events related to negative emotions. Through in-depth understanding of the pathogenesis of AMI, the current treatment methods such as coronary interventional therapy, thrombolysis and coronary artery bypass grafting can effectively improve symptoms and improve survival rate. However, many patients still have fears about the disease and concerns about the treatment. With the global spread of COVID-19, the psychological problems of patients in the post-epidemic era have become more prominent. There are many psychological issues that need to be addressed. Mood disorders such as anxiety or depression can affect the neuroendocrine system. Sympathetic excitation leads to increased catecholamine secretion and coronary spasm, which can delay recovery or even worsen disease progression.

In addition, anxiety or depression may also lead to a decline in patient treatment compliance and clinical treatment efficacy. According to the results of many studies, depression is independently associated with poor prognosis, low life quality in health, disability, recurrent adverse cardiac events, and high mortality in patients with AMI (23). Depression is a risk factor for all-cause mortality, cardiac mortality, and composite outcomes including mortality or non-fatal cardiac events in patients with AMI (24). In hospitalized patients with acute coronary syndrome, depression increases the risk of acute coronary syndrome events. In the COPES study (Coronary Psychosocial Evaluation Study), 157 patients with acute coronary syndrome with persistent depressive symptoms received a 6-month psychological intervention for depression. This intervention significantly improved depression and reduced the incidence of serious adverse cardiac events compared with usual care (25). According to a study of cardiac rehabilitation in patients with coronary heart disease, cardiac rehabilitation combined with stress management training can reduce the incidence of clinical adverse events compared with cardiac rehabilitation alone (26). Patients hospitalized for acute myocardial infarction undergoing percutaneous coronary intervention or coronary bypass surgery in the past 12 months were invited to participate in a trial of psychotherapy. One group of participants received 1 year of cognitive behavioral therapy, while the other group received usual care. Findings showed a 41% reduction in the incidence of fatal and non-fatal first recurrent cardiovascular disease events in the intervention group (27). In a cohort study of the 12-month ESDEPACS trial (Escitalopram in Acute Coronary Syndrome), the incidence of major adverse cardiac events was significantly lower in patients with acute coronary syndrome in the escitalopram group than in the placebo group over an 8.1-year period (28). These studies all suggest that the “psycho-cardiology” model has a better effect than conventional treatment in patients with acute myocardial infarction. Therefore, clinical practice should also pay attention to the psychological problems of patients and conduct early evaluation. When necessary, synergistic optimization of treatment is feasible.

## 5. Conclusion

As suggested by the results of this study, the Psycho-Cardiology is significantly efficacious in improving the anxiety, depression and cardiac function and in reducing the occurrence of adverse cardiovascular events (*P* < 0.05). When managing patients who may be at risk of cardiovascular disease, it is necessary to strengthen the screening of their psychological state (29), especially female patients, who are more prone to depression and have markedly higher mortality of AMI than the male patients (30). For patients with abnormal psychological state, early Psycho-Cardiology treatment should be given, including the psychological intervention, cardiac rehabilitation exercise, and if necessary, antidepressant medication should be given as well (31). Such treatment can help patients deepen awareness of the disease and build confidence to overcome it, which thus improves their quality of life, enhances the long-term efficacy and betters their prognosis.

## Data availability statement

The original contributions presented in the study are included in the article/supplementary material, further inquiries can be directed to the corresponding author.

## Ethics statement

The studies involving human participants were reviewed and approved by Chenzhou No. 1 People Hospital Ethics Committee. The patients/participants provided their written informed consent to participate in this study. Written informed consent was obtained from the individual(s) for the publication of any potentially identifiable images or data included in this article.

## Author contributions

XC determined the research direction. CC and LC participated in the follow-up and collected data. XC and DZ funded the research project. MZ and CC wrote the article and analyzed the data. All authors contributed to the article and approved the submitted version.
